# Comparative data on corrosion protection of mild steel in HCl using two new thiazoles

**DOI:** 10.1016/j.dib.2022.107838

**Published:** 2022-01-17

**Authors:** Ahmed Alamiery, Abu Bakar Mohamad, Abdul Amir H. Kadhum, Mohd S. Takriff

**Affiliations:** Department of Chemical and Process Engineering, Faculty of Engineering and Built Environment, Universiti Kebangsaan Malaysia, Bangi, Selangor 43600, Malaysia

**Keywords:** Corrosion, Mild steel, Inhibitor, Hydrochloric acid

## Abstract

This data article includes data described in the investigation report entitled “The synergistic role of azomethine group and triazole ring at improving the anti-corrosive performance of 2-amino-4-phenylthiazole” (Alamiery et al., 2021). In this data article, a comprehensive effect of 2-Amino-4-phenyl-N-benzylidene-5-(1,2,4-triazol-1-yl)thiazole (APNT) and 2-amino-4-phenylthiazole (APT) and optimized process parameter of the inhibitor in 1 M HCl solution was presented using gravimetric techniques and Density functional theory. The presence of the inhibitors influenced the corrosion resistance of mild steel (MS). Inhibition efficiencies values of 98.1% and 94.74% were recorded as results of inhibition of the MS by the inhibiting compounds APNT and ATP respectively. DFT studies observed that the presence of benzylidene to the APNT and the substitution of a triazole in the thiazole ring are adsorption sites that increase the interaction of the APNT molecules with the iron atoms on the MS surface.

**Specifications Table**

**Table:** 

Subject	Materials science
Specific subject area	Corrosion and surface science
Type of data	Tables and Figures
How the data were acquired	The polished and weighed coupon was immersed in 1 M hydrochloric acid environment of various inhibitors (APNT or APT) concentrations. The pre-weighed MS coupons were exposed to the corrosive environment for 1, 5, 10, 24, and 48 h, washed properly, dried, and weighed.
Description of data collection	Synthesis, analysed
Data source location	Department of Chemical and Process Engineering, Faculty of Engineering and Built Environment, Universiti Kebangsaan Malaysia, Bangi, Selangor 43600, Malaysia.
Data accessibility	Data is within this article
Related research article	*A. Alamiery, A. Mohamad, A. H. Kadhum, M. S. Takriff, The synergistic role of azomethine group and triazole ring at improving the anti-corrosive performance of 2-amino-4-phenylthiazole, South African Journal of Chemical Engineering, 38, (2021), 41-53.*https://doi.org/10.1016/j.sajce.2021.07.003[1]

## Value of the Data

• Data displayed here to afford optimum conditions of phenylthiazole derivatives as inhibitors (APNT and APT) for MS in a 1 M HCl environment. The provided data illustrate the inhibition efficiencies of APNT and APT on MS corrosion in hydrochloric acid solution.
• The data collected for the inhibition of APNT and APT on MS can be applied as a source in evaluating the inhibition efficiencies of the same inhibitors in other corrosive solutions.
• The data can be applied to check the correlation between process variable as it affects the nature of the alloy's inhibition.

## 1 Data Description

**Fig. 1:**
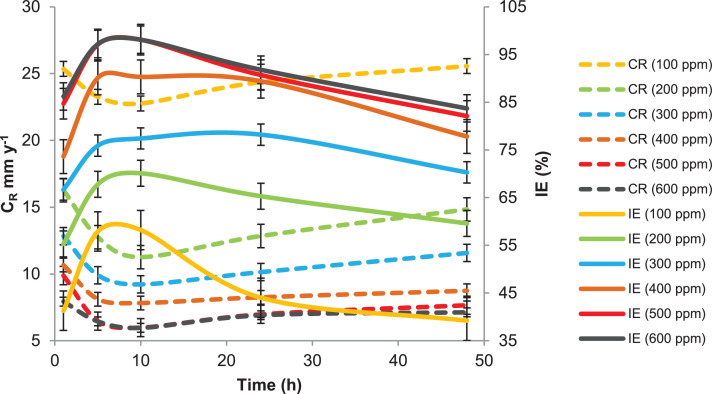
Rate of corrosion and inhibitory efficacy against exposure time for mild steel in 1 M HCl environment in presence of various concentrations of APNT.

**Fig. 2:**
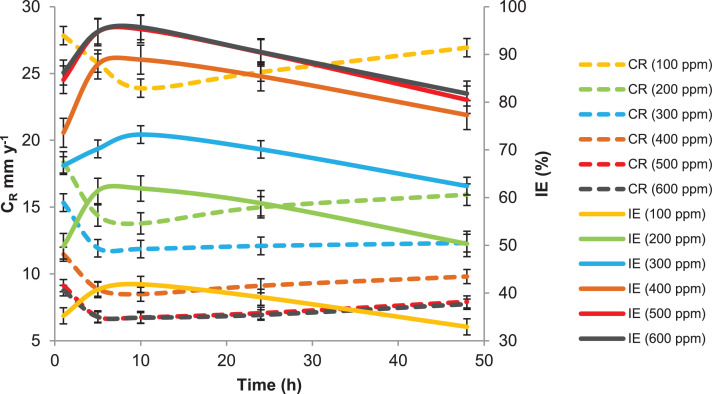
Rate of corrosion and inhibitory efficacy against exposure time for mild steel in 1 M HCl environment in the presence of various concentrations of APT.

**Fig. 3:**
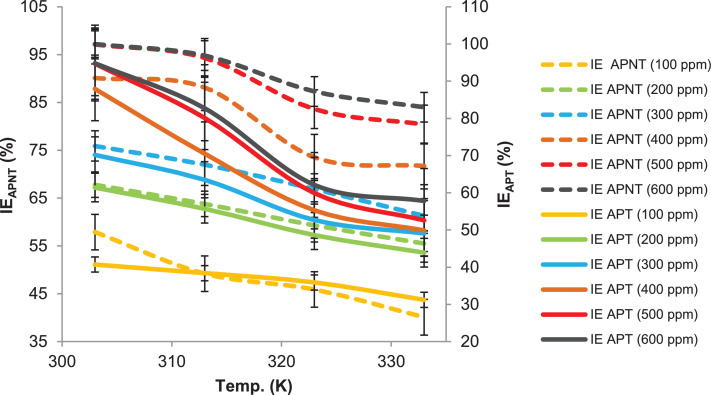
Inhibition efficiencies of various concentrations of APNT and APT vs Temperature for MS in 1 M HCl solution for 5 h as immersion time.

This paper contains data on the chemical structure of two new corrosion inhibitors (APNT and APT). Data obtained from the corrosion protection weight loss measurements of mild steel in a 1 M HCl environment in the absence and presence of different concentrations of tested inhibitors are shown in Figs. 1 and 2, respectively. It can be clearly seen from Figs. 1 and 2 that the inhibition efficiency of the tested inhibitors increases with the concentrations of the inhibitors increase. The increase in the inhibiting efficiencies could be due to the increase in the number adsorbing APNT and APT molecules of onto the surface of mild steel. The most important inhibitory performance was achieved by APNT at the optimum utilized concentration of 500.0 ppm, with an inhibition efficiency of 98%. The highest inhibitory efficiency of APNT was due to the transferring of electron pairs from the azomethine group and triazole ring to the unoccupied d-orbital of Fe atoms on the mild steel surface and forming coordination bonds that control and/or inhibit the corrosion phenomenon. Increasing the concentration of the tested inhibitor by more than 500 ppm does not lead to any noticeable change in the inhibitory efficiency as demonstrated in Figs. 1 and 2, which is related to the saturation of the mild steel surface with the inhibitor molecules. It is further noted that temperature is an important parameter for the inhibition efficiencies of each of the tested inhibitors. For APNT, the value of the inhibition efficiency remains nearly constant as the temperature rises from 303 K to 323 K, but a significant decrease is observed when the temperature rises to 333 K (Fig. 3). Moreover, at the optimum concentration of the APT, the inhibition efficiency decreases with increasing temperature and as shown in Fig. 3. We also note from Fig. 3 that the inhibition efficiency of APNT decreases from 97.1 to 80.4% and compared with the other inhibitor (APT), the inhibition efficiency decreases sharply from 94.1 to 52.6% in the temperature range 303 K to 333 K. This characteristic reveals that as the temperature increases, the inhibitor molecules are desorbed from the mild steel surface, and become unprotected by the inhibitor molecules, thus reducing the inhibition efficiency.

**Fig. 4:**
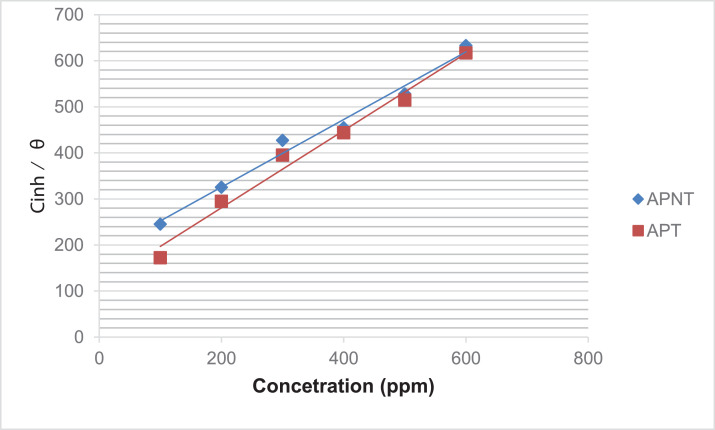
Langmuir adsorption model plots for APNT and APT.

The surface coverage (θ) is quite important for considering the adsorption properties. The dependence of the surface coverage on the inhibitor concentration (C) was examined graphically by fitting it to Langmuir's isotherm. Fig. 4 exhibits the linear plots for C/θ aginst C, implying that the adsorption isotherm follows Langmuir's adsorption isotherm.

The negative values of Gibbs free energy $\left(\Delta G_{\text{ads}}^{o}\right)$ indicate the spontaneous adsorption of inhibitors on the surface of mild steel [2]. In the present study, $\Delta G_{\text{ads}}^{o}$ values for APNT and APT are $-39.7,\;\text{and}-37.2\;\text{kJ}\;\text{mo}l^{-1}$, respectively indicating that the adsorption is the combination of both physisorption and chemisorption [3], [4], [5].

To determine the active sites of the inhibitor molecule, two influence parameters: frontier molecular orbitals, and electronic are considered [6]. According to classical chemical theory, all chemical interactions are either electrostatic or orbital. [7,8]. The electronic parameters are calculated according to Koopmans's theorem [9] and it was found that the total energy values were evaluated 2.0795 eV and 0.03427 eV for APNT and APT respectively. A quick look at the EHOMO (–9.002 eV and –9.104 eV) values and LUMO values (–1.807 eV and –2.023 eV) for APNT and APT (Table 1) respectively reveals that these two compounds have the same susceptibility. The order of HOMO energy is as follows:

APNT>APT.

**Table 1:** E_HOMO_-E_LUMO_ values for APNT and APT.

Inh.	E_HOMO_	E_LUMO_	E_HOMO-1_	E_LUMO+1_	E_HOMO-2_	E_LUMO+2_
APNT						

	-9.002 eV	-1.807 eV	-10.532 eV	-1.060 eV	-11.267 eV	-1.045 eV

		

APT						

	-9.104 eV	-2.023 eV	-9.262 eV	-1.057 eV	-12.109 eV	+4.577 eV

		

The process of receiving electrons is essential to LUMO energy. A low LUMO energy indicates that the inhibitor molecules can find another negative charge on the mild steel surface. The order of LUMO energy is as follows:

APNT>APT.

APNT molecules are most effective in inhibiting corrosion of mild steel according to EHOMO-ELUMO. (Table 1).

## 2 Experimental Design, Materials and Methods

### 2.1 Weight loss analysis

This physical analysis was conducted to present straight results on how the acidic solution affects the examine samples. The polished, dried, and weighed coupon was suspended in glasses with the support of a glass hook and rod with the examine solutions of APNT (or APT) at various concentrations (100, 200, 300, 400, 500, and 600 ppm). The pre-weighed coupon was retrieved from the examine solutions after every 1, 5, 10, 24, and 48  h, cleaned and weighed. The variation between the mass at a presented time and the original mass of the coupon were considered as the mass loss that was utilized to determine the rates of corrosion (Eq. 1) and protection efficiencies (Eq. 2). The effect of temperature on the corrosion phenomenon was achieved out at three various temperatures: 313, 323, and 333 K [10,11].

(1) $$C_{R}=\frac{87.6w}{atd}$$

(2) $$IE\;\%=\frac{w_{o}-w_{i}}{w_{o}}\times 100$$

where *w* is the MS mass loss (mg), *a* is the MS coupon area ($cm^{2})$, *t* is exposure period (hrs), and *d* is the coupon density ($g/cm^{3}),$ $w_{o}$ is MS coupon mass loss, $w_{i}$, is MS coupon mass loss with various concentrations of tested inhibitors.

### 2.2 DFT analysis

Density Functional Theory (DFT) approach are able to optimise molecule structures and calculate the corrosion inhibitor efficiency quantum parameters such as HOMO, LUMO, electron affinity and ionization potential. Gaussian 03, Revision C.01 [12], was optimized to a local optima without symmetry restrictions (6-31G++(d,p)) [13], [14], [15], [16], [17], [18], [19], [20]. All the calculations were used to determine important chemical parameters. This technique was successfully applied to describe the phenol structural of corrosion inhibitors and efficiency on mild steel surface.

## CRediT Author Statement

**Ahmed Alamiery:** Conceptualization, Methodology, Software **Mohd S.Takriff**: Data curation, Writing- Original draft preparation. **Abdul Amir H. Kadhum**: Visualization, Investigation. **Abu Bakar Mohamad:** Supervision. **Mohd S.Takriff**: Software, Validation.: **Ahmed Alamiery:** Writing- Reviewing and Editing.

## Declaration of Competing Interest

The authors declare that they have no known competing financial interests or personal relationships that could have appeared to influence the work reported in this paper.
